# COVID-19 Related Acute Hemorrhagic Necrotizing Encephalitis: A Report of Two Cases and Literature Review

**DOI:** 10.7759/cureus.14236

**Published:** 2021-04-01

**Authors:** Naresh Mullaguri, Sanjeev Sivakumar, Anusha Battineni, Samyuktha Anand, Joshua Vanderwerf

**Affiliations:** 1 Medicine/Neurological Intensive Care, Prisma Health Greenville Memorial Hospital, Greenville, USA; 2 Medicine/Neurology, University of South Carolina, Greenville, USA; 3 Neurology, Prisma Health Greenville Memorial Hospital, Greenville, USA

**Keywords:** sars-cov-2 (severe acute respiratory syndrome coronavirus -2), acute hemorrhagic necrotizing encephalitis, covid-19, cerebral microhemorrhage, disorder of consciousness, dexamethasone convalescent plasma, remdesivir, cytokine release storm

## Abstract

Coronavirus disease 2019 (COVID-19) caused by severe acute respiratory syndrome coronavirus 2 (SARS-CoV-2), a novel coronavirus, has proven neurotropism and causes a multitude of neurologic manifestations. Acute hemorrhagic necrotizing encephalitis (AHNE), though rare, can be seen in patients with severe infection and is associated with devastating neurologic outcomes. The true prevalence of this syndrome is unknown due to underrecognition, difficulty in timely acquisition of neuroimaging, and high mortality in this subset of patients escaping detection. It is a distinct clinicoradiological syndrome, with patients suffering from rapidly worsening encephalopathy and coma within the first two weeks of severe illness and hemorrhagic necrotizing parenchymal changes on neuroimaging. The pathophysiology of this syndrome is unclear but hypothesized to occur due to cytokine storm, blood-brain-barrier dysfunction, and direct viral-mediated endotheliopathy. Diagnosis requires a high index of suspicion in patients who have unexplained persistent severe encephalopathy associated with COVID-19 infection. Most patients have elevated systemic inflammatory markers and severe lung disease with hypoxic respiratory failure requiring mechanical ventilation. MRI is the imaging modality of choice, with a distinct neuroimaging pattern. CSF (cerebrospinal fluid) studies have a low yield for viral particle detection with currently available testing. While long-term outcomes are unclear, early immunomodulatory treatment with intravenous immunoglobulin, plasma exchange, and steroids may portend a favorable outcome.

We discuss two cases of COVID-19 related AHNE and also include a pertinent literature search of similar cases in PubMed to consolidate the AHNE clinical syndrome, neuroimaging characteristics, management strategies, and reported short-term prognosis.

## Introduction

Severe acute respiratory syndrome coronavirus 2 (SARS-CoV-2) has been shown to use the angiotensin-converting enzyme 2 (ACE2) receptors for entry into host cells [1]. The expression of ACE2 receptors in the endothelium, glial cells, and neurons makes the brain a potential target of COVID-19 [2]. Neurotropism may occur via the upper nasal cribriform route or via blood circulation, allowing COVID-19 to reach the brain, and bind and engage with ACE2 receptors [3]. Patients with acute SARS-CoV illness have shown the presence of SARS-CoV-2 virus in cerebrospinal fluid (CSF). Neurotropism is highlighted by numerous reports on neurological manifestations, with a prevalence as high as 35%, among hospitalized patients with COVID-19 [4,5]. Manifestations include headache, myalgia, olfactory disorders, meningoencephalitis, myelitis, necrotizing encephalopathy, impaired consciousness, stroke, seizures, Guillain-Barré syndrome, and acute demyelinating encephalomyelitis [5-7]. Many of the coronavirus disease 2019 (COVID-19) related post-infectious inflammatory neurological conditions, such as acute disseminated encephalomyelitis, acute inflammatory demyelinating neuropathy, and acute necrotizing hemorrhagic encephalopathy, mirror those seen with other viral respiratory diseases and coronaviruses [8]. Acute hemorrhagic necrotizing encephalitis (AHNE) due to COVID-19 is a rare but disabling post-infectious inflammatory condition of the central nervous system. Cytokine storm syndrome with a resultant dysregulation of the blood-brain-barrier is a proposed mechanism of AHNE [2,3]. An acute and rapidly progressive encephalopathy, including hemorrhagic necrosis of the parenchyma and associated high mortality, is typical of AHNE [9,10]. Characteristic findings on MRI of the brain in AHNE patients include symmetrical T2/FLAIR (T2-weighted/fluid-attenuated inversion recovery) hyperintense lesions involving the cortex, subcortical white matter, basal ganglia, thalami, brain stem, and cerebellar hemispheres, along with diffuse microhemorrhages on susceptibility-weighted imaging [11,12]. Limited reports in the literature describe AHNE incidence and outcomes among patients admitted with COVID-19 [11-18]. In this report, we present two cases of AHNE in the setting of severe COVID-19 infection detailing findings on neuroimaging and a brief review summarizing other reports of COVID-19 associated AHNE.

## Case presentation

Patient 1

**Figure 1:**
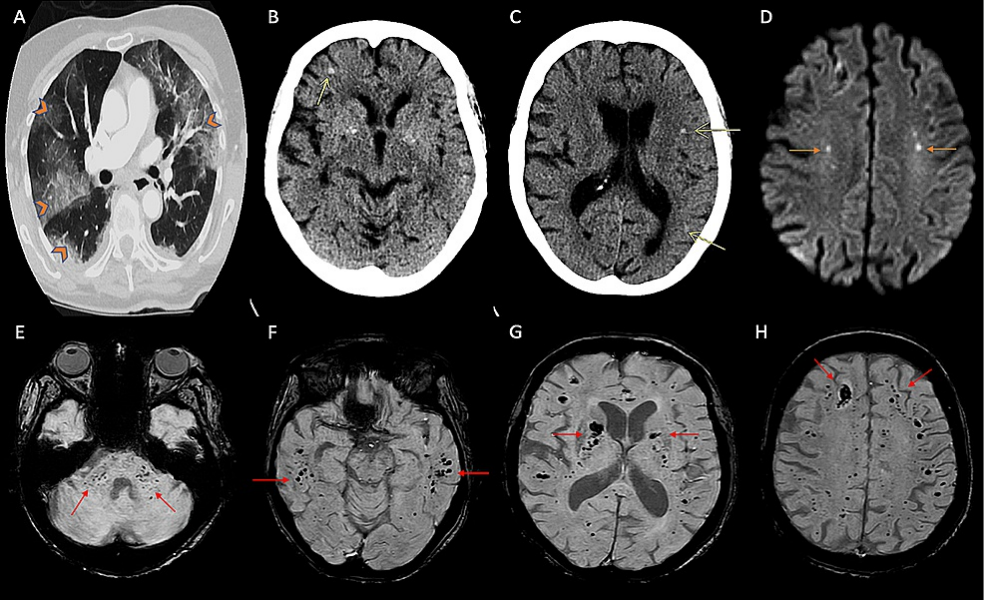
Imaging findings of patient 1 (A) CT (axial section) of the chest with contrast showing diffuse bilateral subpleural and perihilar ground-glass opacities (orange arrowheads). (B, C) CT head (axial sections) of the brain showing punctate hemorrhages in the right frontal and left frontal and parietal areas (yellow arrows). (D) MRI of the brain (axial section, diffusion-weighted imaging) showing hyperintensities in bilateral centrum semiovale areas (orange arrows). (E-H) MRI of the brain (susceptibility-weighted imaging) showing innumerable punctate microhemorrhages in the cerebellar peduncles and subcortical regions of bilateral hemispheres including bilateral basal ganglia and internal capsules (red arrows). CT, computed tomography; MRI, magnetic resonance imaging

A 77-year-old Caucasian woman with Parkinson's disease, cognitive impairment, and hypertension presented to the emergency room with fever, fatigue, disorientation, and progressive shortness of breath. She was a former five-pack-year smoker who quit 50 years ago. On initial evaluation, she had a temperature of 37.8°C, respiratory rate of 31 bpm, tachycardic at 114 bpm, blood pressure of 157/79 mmHg, and oxygen saturation of 85% on room air. She was oriented to self but not to place or time; otherwise, her neurologic examination was unremarkable. Auscultation of her chest revealed bilateral coarse rales in both lungs. She required intubation and mechanical ventilation for severe hypoxic respiratory failure. Laboratory workup showed normal white cell count (11.0 thousand/mm^3^), hyponatremia (132 mMol/L), significant elevations in D-dimer (>20 ug/mL), lactate dehydrogenase (450 u/L), ferritin (646 ng/mL), C-reactive protein (203 mg/L), and creatine kinase (338 IU/L), with a subsequent upward trend in the aforementioned inflammatory markers. Real-time reverse-transcriptase polymerase chain reaction (RT-PCR) assay of nasopharyngeal swabs returned positive for SARS-CoV-2. Computed tomography (CT) of the chest (Figure 1A) demonstrated extensive bilateral ground-glass pulmonary opacities with dense consolidation in the right greater than the left lower lobe. She was treated with low tidal volume positive pressure ventilation, a 10-day course of dexamethasone, a five-day course of remdesivir, and convalescent plasma. The patient was comatose with no response to central or peripheral noxious stimulation except intact bilateral pupillary light reflex, corneal, and cough reflex. A non-contrast CT scan of the head on day 10 of hospitalization showed several small parenchymal hemorrhages in bilateral cerebral hemispheres, involving the frontoparietal and temporal lobes (yellow arrows, Figures 1B, 1C). CT angiography of the head was unremarkable for hemodynamically significant atherosclerosis or vasculopathy. MRI of the brain demonstrated tiny foci of restricted diffusion involving bilateral centrum semiovale (Figure 1D) and inferior left cerebellar hemisphere. Susceptibility-weighted imaging revealed innumerable areas of microhemorrhages in the bilateral cerebral hemispheres involving the corona radiata, centrum semiovale, internal capsule, globus pallidus, the gray-white junction of all lobes, pons, bilateral middle cerebellar peduncles, and cerebellar hemispheres (Figures 1E-1H). These findings were concerning for AHNE in the setting of severe COVID-19 infection. MRI of the brain obtained five years ago for cognitive impairment did not show any microhemorrhages. Twenty-eight days into hospitalization, she remained comatose. Her oxygen requirement escalated to 100% on mechanical ventilation. The patient subsequently died following the humane withdrawal of life support measures.

Patient 2

**Figure 2:**
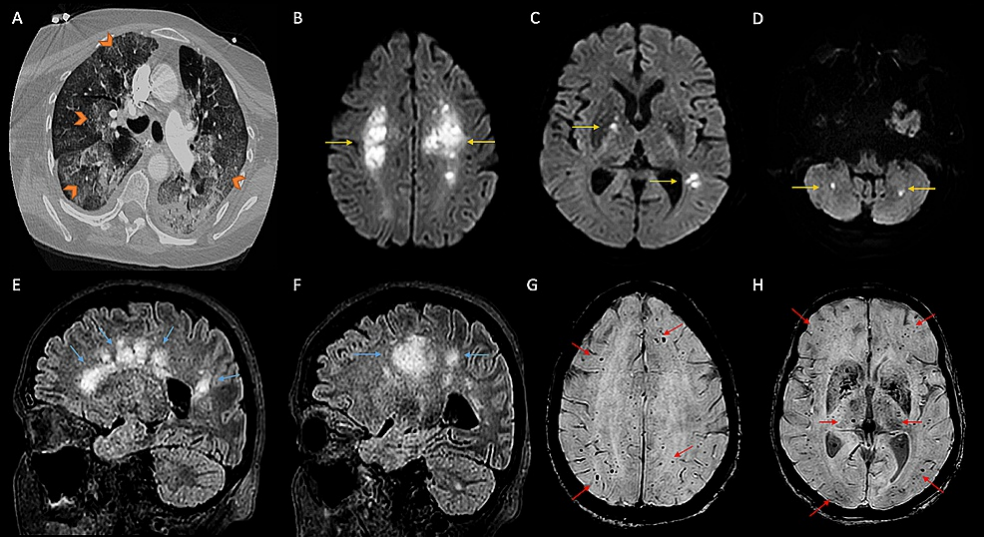
Imaging findings of patient 2 (A) CT (axial section) of the chest with contrast showing diffuse bilateral subpleural and perihilar ground-glass opacities (orange arrowheads). (B-D) MRI of the brain (diffusion-weighted imaging, axial sections) showing hyperintensities in bilateral centrum semiovale, basal ganglia, and bilateral cerebellar hemispheres (yellow arrows). (E, F) T2/FLAIR sagittal images showing confluent hyperintensities in periventricular regions, centrum semiovale in frontoparietal areas, and cerebellum (blue arrows). (G, H) Susceptibility-weighted imaging showing multiple foci of microhemorrhages in cortical and subcortical regions, and bilateral basal ganglia (red arrows). CT, computed tomography; MRI, magnetic resonance imaging; T2/FLAIR, T2-weighted/fluid-attenuated inversion recovery

A 68-year-old woman presented to the emergency room with malaise, nausea, diarrhea, progressive dyspnea, and high-grade fever. Her medical history was significant for chronic lymphocytic leukemia and hypertension. Her husband had recently died from severe SARS-CoV2 associated pneumonia. Five days before her acute presentation, she tested positive for COVID-19 based on a positive result on RT-PCR assay of the nasopharyngeal swab. She was febrile at 39.4°C and tachypneic, with oxygen saturation of 88% on room air on examination. Chest auscultation revealed bilateral coarse rales. The remainder of her physical examination was unremarkable. She required endotracheal intubation and mechanical ventilation for hypoxic respiratory failure. Laboratory workup showed lymphocytic predominant leukocytosis (23.3 thousand/mm^3^) (83% lymphocytes). Systemic inflammatory markers such as D-dimer (0.5 ug/mL), lactate dehydrogenase (295 u/L), ferritin (259 ng/mL), and C-reactive protein (177 mg/L) were elevated with a subsequent upward trend. Figure 2 shows the finding of imaging. CT of the chest (Figure 2A) demonstrated extensive bilateral ground-glass pulmonary opacities that appeared more prominent in the periphery of both lungs' upper lobes. She was treated with low tidal volume positive-pressure ventilation, a 10-day course of dexamethasone, a five-day course of remdesivir, and convalescent plasma. Neurologic consultation was obtained for persistent severe encephalopathy on day 30 of hospitalization. She was comatose with no responses to painful stimulation while on mechanical ventilation, with preserved bilateral pupillary light reflex, normal corneal, and cough reflex. A non-contrast CT scan of the head showed patchy bilateral white matter hypodensities concerning age indeterminate infarcts. Continuous electroencephalography was unable to be performed due to the patient's acute renal failure requiring sustained low-efficiency dialysis due to interference and artifact. MRI of the brain demonstrated multifocal T2/FLAIR hyperintense periventricular lesions (Figures 2E, 2F). The same regions showed diffusion restriction involving the bilateral centrum semiovale (Figure 2B), right internal capsule, left parietal cortex (figure 2C), and bilateral cerebellum (Figure 2D). Susceptibility-weighted imaging sequence demonstrated multiple areas of susceptibility artifact consistent with microhemorrhages in the bilateral cerebral cortex, basal ganglia, and cerebellar hemispheres (Figures 2G, 2H). These findings were most consistent with the diagnosis of AHNE in the setting of severe COVID-19 infection. Apart from severe acute respiratory failure, she was treated with vasopressors for septic shock due to COVID-19 and hemodialysis for acute renal failure. Fifty-four days into hospitalization, the patient remained unresponsive to tactile and noxious stimulation. Her clinical condition deteriorated and subsequently died from refractory hypoxia, shock, and cardiac arrest.

## Discussion

While SARS-CoV-2 primarily causes respiratory disease, the central and peripheral nervous system's involvement is not infrequent. Meningitis, encephalitis, seizures, ischemic and hemorrhagic stroke, and persistent disorders of consciousness are among frequently reported central nervous system manifestations of COVID-19 illness [5-8,11-18]. AHNE is a rare neurologic complication secondary to para-infectious and hyperimmune response to SARS-CoV-2 infection. This clinicoradiological syndrome affects patients with severe COVID-19 infection and occurs between one to two weeks after the onset of the upper respiratory tract infection. The true incidence is unknown due to underrecognition of the syndrome and difficulties in obtaining timely neuroimaging studies due to patients' disease severity.

The pathophysiology of COVID-19 related AHNE is unclear. Several mechanisms were hypothesized, including hypercoagulable state from systemic inflammation, cytokine storm, post-infectious immune-mediated responses, direct viral-induced endotheliopathy leading to angiopathy, and microthrombosis [1-3]. Viral particles have been isolated from the endothelium of various tissues, including the brain [2,3]. The causal mechanisms for widespread cerebral microhemorrhage due to SARS-CoV2 infection is unclear. The viral particle-induced endothelial injury could be one plausible mechanism. Cytokine storm-mediated IgG (immunoglobulin G) production and breakdown of blood-brain-barrier are likely contributory mechanisms [19,20]. The effect of various treatment modalities such as steroids, polyvalent immunoglobulins, and other immunotherapy on the clinical course and long-term outcomes among patients diagnosed with AHNE secondary to COVID-19 needs evaluation in larger prospective cohorts.

Our literature review in PubMed until September 2020 for COVID-19 patients with AHNE identified 23 patients between the ages of 43 and 77 years, with a median age of 58 years. More than 50% had medical comorbidities such as hypertension and diabetes mellitus, and only one patient had a prior neurologic disease (Table 1) [11-16].

**Table 1:** List of published cases of SARS-CoV-2 related acute hemorrhagic necrotizing encephalitis: clinical characteristics, neuroimaging findings, treatment, and outcomes CAD, coronary artery disease; CLL, chronic lymphocytic leukemia; CSF, cerebrospinal fluid; DM, diabetes mellitus; DM, diabetes mellitus; FLAIR, fluid-attenuated inversion recovery; GFAP, glial fibrillary acidic protein; HLD, hyperlipidemia; HTN, hypertension; IgG, immunoglobulin G; IL-6, interleukin-6; IVIG, intravenous immunoglobulin; MRI, magnetic resonance imaging; MRI, magnetic resonance imaging; MV, mechanical ventilation; OCB, oligoclonal bands; PCR, polymerase chain reaction; SARS-CoV-2, severe acute respiratory syndrome coronavirus 2; SWI, susceptibility-weighted imaging

Publication	Age/sex	Medical comorbidities	Neurological comorbidities	Presenting symptoms	Neurological symptoms	CSF	Interval between presentation and neuroimaging	Neuroimaging	Treatment	Clinical outcome
Poyiadji et al., 2020 [11]	58/F	-	-	Fever, cough	Altered mental status	Traumatic tap, SARS-CoV-2 not tested	-	CT: bilateral medial thalami hypoattenuation; MRI: hemorrhagic rim-enhancing lesions in the thalami, medial temporal lobes, subinsular regions	IVIG	-
Dixon et al., 2020 [13]	59/F	Aplastic anemia	-	Abdominal pain, diarrhea, cough, dyspnea, headache, myalgia	Recurrent complex partial seizures with secondary generalization, encephalopathy, extensor plantar response, and absent left pupillary response	Opening pressure of 28 cm H_2_O, increased protein, CSF SARS-CoV-2 negative	1 day	CT: brain stem swelling, hypodensity in the left occipital lobe, symmetric hypodensities in deep grey structures and amygdala, pontine hemorrhage; MRI: swelling and hemorrhage in brainstem, amygdala, diffusion restriction, swelling, microhemorrhages, and peripheral enhancement in bilateral basal ganglia, subinsula, splenium of corpus callosum, cingulate gyri, corona radiate, basal cistern effacement, ventricular effacement, uncal and tonsillar herniation	High-dose Dexamethasone	Withdrawal of care on 20th day
Virhammar et al., 2020 [14]	55/F	-	-	Fever, myalgia	Encephalopathy, unresponsiveness, multifocal myoclonus	Mildly increased IgG and OCBs; marked increase in Neurofilament light and tau (biomarkers of neuronal injury), marked elevation of GFAP (biomarker of astrocytic activation and neuroinflammation); mildly elevated IL-6 level; proteomics: neuronal rescue proteins markedly elevated; SARS-CoV-2 N-gene was detected in low concentrations (third week; commercial PCR assays did not detect)	1 day	CT: symmetric thalamic hypodensities; midbrain MRI (day 12): symmetric T2 hyperintense lesions in subinsular regions, medial temporal lobes, hippocampi, cerebral peduncles, thalami, and brain stem with cytotoxic edema and contrast enhancement; petechial hemorrhages in bilateral thalami and subinsular regions in SWI sequence MRI (day 19) - substantial decrease in hyperintensities in hippocampi and mesencephalon; increased contrast enhancement, petechial hemorrhages unchanged	IVIG on day 7; plasma exchange on day 20; convalescent plasma	Improved neurological exam; discharged to rehabilitation on day 35
Radmanesh et al., 2020 [12]	56/M	-	-		Altered sensorium			MRI (day 17 of MV): leukoencephalopathy and microhemorrhages		-
	45/M	Hyperlipidemia	-		Altered sensorium			MRI (day 23 of MV): microhemorrhages		-
	60/M	-	-		Altered sensorium			MRI: leukoencephalopathy and microhemorrhages		-
	43/M	-	-		Altered sensorium			MRI: leukoencephalopathy		-
	64/M	HTN, HLD, DM, CAD	-		Altered sensorium			MRI: leukoencephalopathy and microhemorrhages		-
	63/F	HTN,HLD, DM	-		Altered sensorium			MRI: leukoencephalopathy and microhemorrhages		-
	49/M	HTN, DM	-		Altered sensorium			MRI: leukoencephalopathy		-
	38/M	HTN, DM, cocaine use	-		Altered sensorium			MRI: leukoencephalopathy and microhemorrhages		-
	43/M	-	-		Altered sensorium			MRI: leukoencephalopathy		-
	64/M	HTN, AF	-		Altered sensorium			MRI: leukoencephalopathy and microhemorrhages		-
	62/F	HTN, DM, obesity	-		Altered sensorium			MRI: leukoencephalopathy		-
Paterson et al., 2020 [15]	66/F	-	-	-		Elevated protein, OCB, SARS-CoV-2 negative		MRI: T2 hyperintensities in pons, thalami, subcortical white matter, and limbic regions	Methylprednisolone, IVIG	Incomplete
	52/M	-	-	-		Unremarkable		MRI: multiple clusters of deep cerebral white matter lesions; cyst-like areas of variable sizes with some hemorrhagic foci; peripheral rims of restricted diffusion		Incomplete
	60/M	-	-	Cough , fever, dyspnea, myalgia		SARS-CoV-2 negative		MRI: multifocal and confluent areas of signal change in the white matter; extensive microhemorrhages in the subcortical regions	Methylprednisolone	Incomplete
	59/F	-	-			Elevated opening pressure, SARS-CoV-2 negative		MRI: extensive, confluent largely symmetric areas in the brainstem, limbic regions, insular lobes, subcortical white matter and deep grey structures; clusters of microhemorrhages, restricted diffusion, and peripheral rim enhancement	Dexamethasone	Expired
	52/M	-	-	Headache, back pain, vomiting	Bilateral face, neck, upper and lower extremity weakness with preserved sensation	Elevated protein		MRI: multifocal confluent lesions in internal and external capsules, splenium, subcortical white matter; multiple microhemorrhages and extensive prominent medullary veins; brachial and lumbosacral plexus hyperintensities and contrast enhancement	Methylprednisolone; IVIG	Incomplete recovery; able to follow commands
	47/F	-	-	Cough, fever, dyspnea	Right-sided headache, left-sided numbness, and weakness	Unremarkable, brain biopsy was negative for SARS-CoV-2		CT: right hemispheric vasogenic edema with midline shift; MRI: severe right hemispheric vasogenic edema. mass effect with 1-cm midline shift to the left, small T2 hyperintense lesions in the left hemisphere	Methylprednisolone, decompressive hemicraniectomy, prednisone, IVIG	Incomplete, weight bearing with support
Scullen et al., 2020 [16]	43/F	HTN, DM		Cough, dyspnea	Left hemiparesis and extensor posturing on the right side, severe encephalopathy		Day 15	MRI: hyperintensities in bilateral mesial temporal lobes, lenticular nuclei, crus cerebri, centrum semiovale in FLAIR sequence; restricted diffusion in the same regions along with splenium, body and genu of corpus callosum; hemorrhagic conversion in the left cerebral peduncle, bilateral basal ganglia.	Plasma exchange	incomplete
Mullaguri et al., 2021 (present study)	77/F	HTN	Parkinson’s disease	Fever, fatigue, confusion and dyspnea	Persistent severe encephalopathy		Day 10	CT: bilateral frontotemporal and parietal lobe hemorrhages; MRI: tiny foci of restricted diffusion involving bilateral centrum semiovale and inferior left cerebellar hemisphere; SWI sequence showed innumerable hypointense foci in the bilateral cerebral hemispheres involving the corona radiata, centrum semiovale, internal capsule, globus pallidus, the gray-white junction of all lobes, pons, bilateral middle cerebellar peduncles, and cerebellar hemispheres	Dexamethasone, convalescent plasma. and remdesivir	Dead; remained unresponsive until death
	68/F	HTN, CLL		Malaise, nausea, diarrhea, dyspnea, fever	Persistent severe encephalopathy		Day 30	CT: patchy bilateral white matter hypodensities concerning for age indeterminate infarcts; MRI: multifocal T2-hyperintense periventricular lesions on T2 FLAIR sequence, diffusion restriction involving the bilateral centrum semi ovale, right internal capsule, left parietal cortex, and bilateral cerebellum; SWI sequence demonstrated numerous hypointense foci consistent with microhemorrhages affecting the bilateral cerebral cortex, basal ganglia, and cerebellar hemispheres.	Dexamethasone, convalescent plasma and remdesivir	Dead; remained unresponsive until death

The most common presenting symptoms with SARS-CoV-2 infection were fever, malaise, headache, dyspnea, diarrhea, cough, and abdominal pain, which led to testing. Most patients had severe pulmonary manifestations leading to hypoxemic respiratory failure requiring intubation and mechanical ventilation with multiorgan dysfunction and cytokine storm. Neurologic manifestations of coma and persistent encephalopathy dominated clinical presentation, followed by seizures and focal deficits. The interval between disease onset and neurologic manifestation or neurologic consultation ranged between 1 and 30 days (extrapolated from the neuroimaging data). Based on the available laboratory data, spinal fluid analyses frequently revealed elevated opening pressure, CSF protein, and negative SARS-CoV-2 PCR results. Neuroimaging findings in most patients showed patchy bilateral periventricular hypoattenuation on CT. MRI imaging showed multifocal diffusion restriction, periventricular confluent T2/FLAIR hyperintensities, and diffuse microhemorrhages like in our patients. All patients with AHNE received dexamethasone, with or without immunomodulation therapies such as intravenous immunoglobulin, plasma exchange, or convalescent plasma. Though outcome data were very limited, we found that the recovery rate favored those treated with immunomodulation and dexamethasone than those treated with dexamethasone alone.

## Conclusions

AHNE is a disabling neurologic manifestation of COVID-19. This clinicoradiological condition is characterized by rapidly progressive encephalopathy, with distinct neuroimaging features of multifocal diffusion restriction and microhemorrhages involving the cortex, subcortical and periventricular white matter, brain stem, and infratentorial regions. AHNE diagnosis must be considered in patients with severe COVID-19 infection, with a persistent encephalopathy or coma. Larger cohorts are required to understand better the incidence of AHNE, its association with chronic neurological conditions, and prior immune suppression, therapeutics, and long-term functional outcomes.
